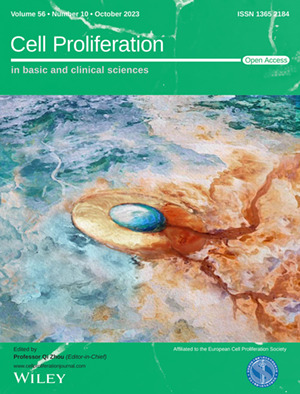# Featured Cover

**DOI:** 10.1111/cpr.13559

**Published:** 2023-10-01

**Authors:** Peng Lin, Pulin Yan, Jun Zhu, Sha Huang, Zhong Wang, Ou Hu, Huaijian Jin, Yangyang Li, Liang Zhang, Jianhua Zhao, Lin Chen, Bing Liu, Jian He, Yibo Gan, Peng Liu

## Abstract

The cover image is based on the Original Article *Spatially multicellular variability of intervertebral disc degeneration by comparative single‐cell analysis* by Peng Lin et al., https://doi.org/10.1111/cpr.13464.